# Multimodal phantoms for clinical PET/MRI

**DOI:** 10.1186/s40658-021-00408-0

**Published:** 2021-08-26

**Authors:** Eve Lennie, Charalampos Tsoumpas, Steven Sourbron

**Affiliations:** 1 Department of Infection, Immunity and Cardiovascular Disease, University of Sheffield, Sheffield, UK; 2 Biomedical Imaging Science Department, University of Leeds, Leeds, UK; 3 Department of Nuclear Medicine and Molecular Imaging, University Medical Center Groningen, University of Groningen, Groningen, The Netherlands

**Keywords:** Positron emission tomography, Magnetic resonance imaging, Phantoms, Hybrid imaging

## Abstract

Phantoms are commonly used throughout medical imaging and medical physics for a multitude of applications, the designs of which vary between modalities and clinical or research requirements. Within positron emission tomography (PET) and nuclear medicine, phantoms have a well-established role in the validation of imaging protocols so as to reduce the administration of radioisotope to volunteers. Similarly, phantoms are used within magnetic resonance imaging (MRI) to perform quality assurance on clinical scanners, and gel-based phantoms have a longstanding use within the MRI research community as tissue equivalent phantoms. In recent years, combined PET/MRI scanners for simultaneous acquisition have entered both research and clinical use. This review explores the designs and applications of phantom work within the field of simultaneous acquisition PET/MRI as published over the period of a decade. Common themes in the design, manufacture and materials used within phantoms are identified and the solutions they provided to research in PET/MRI are summarised. Finally, the challenges remaining in creating multimodal phantoms for use with simultaneous acquisition PET/MRI are discussed. No phantoms currently exist commercially that have been designed and optimised for simultaneous PET/MRI acquisition. Subsequently, commercially available PET and nuclear medicine phantoms are often utilised, with CT-based attenuation maps substituted for MR-based attenuation maps due to the lack of MR visibility in phantom housing. Tissue equivalent and anthropomorphic phantoms are often developed by research groups in-house and provide customisable alternatives to overcome barriers such as MR-based attenuation correction, or to address specific areas of study such as motion correction. Further work to characterise materials and manufacture methods used in phantom design would facilitate the ability to reproduce phantoms across sites.

## Introduction

Positron emission tomography (PET) and magnetic resonance imaging (MRI) are both well-established clinical imaging modalities. PET images are formed by detecting the annihilation photons of positrons emitted by a radioactive tracer administered to patients [1]. These are considered functional images, as tracers are targeted to a particular physiological process, and the amount of radiation detected is proportional to the uptake of tracer in a region. Measures such as standardised uptake value (SUV) allow the radiotracer uptake of a region to be quantified. MRI detects a radiofrequency (RF) signal emitted by protons excited by RF pulses in a strong external magnetic field [2]. This can produce high-resolution anatomical images with high contrast between different soft tissues. In recent years, combined PET/MRI scanners have been released by manufacturers and are entering clinical use. These scanners allow for the simultaneous acquisition of PET and MRI data resulting in combined images from both modalities, with proposed advantages in imaging for a range of clinical areas [3].

In both PET and MRI, test objects known as phantoms are used for scanner performance testing and monitoring, verification of new image acquisition protocols and reconstruction methods, standardisation across equipment and other experimental work. PET phantoms are typically solid vessels of various sizes and geometry filled with different concentrations of radiotracer solution [4]. For quality assurance and performance testing, these would typically be a single container filled with fluid to provide a uniform image, or larger acrylic containers with inserts of simple geometries such as cylinders or spheres. Anthropomorphic phantoms, with cavities which appear to match anatomical geometries in PET images, are used to simulate radiotracer uptake in a specific organ, often against a lower activity background.

MRI phantoms adhere to similar designs for performance testing and are often filled with a highly MRI-visible fluid such as nickel chloride or manganese chloride solution, as used in the phantom developed by the National Institute of Standards and Technology (NIST) and the International Society for Magnetic Resonance in Medicine (ISMRM), the NIST/ISMRM system phantom [5]. Anthropomorphic phantoms in MRI often use gels such as agar to achieve MRI relaxation properties close to human tissues [6]. In these existing forms, neither PET nor MRI phantoms are compatible for imaging with the other modality to take advantage of the simultaneous acquisition available with a combined PET/MRI scanner, simply due to the difference in radiological properties required.

Valladares et al. [7] compared the quality assurance programs for PET/MRI scanners of 8 sites across Europe and found significant variation in approaches. The authors recommend a regime in line with available guidelines such as the National Electrical Manufacturers Association’s (NEMA) report NEMA NU-2 [8] for PET and the American Association of Physicists in Medicine (AAPM) Report 028 for MRI [9]. This satisfactorily covers performance monitoring of scanners, but still leaves both modalities tested individually. This doesn’t reflect clinical use of the scanners and in particular doesn’t allow for a complete assessment of the image reconstruction process when using MRI-based attenuation maps with PET data. It also raises the question as to how phantoms have been used in the field of PET/MRI to date, and to what extent phantoms have been developed that are compatible for simultaneous PET/MRI.

This article examines publications between 2011 and 2021 to identify the phantoms used and developed by institutions working with clinical PET/MRI scanners. In order for a publication to be included in this review, the utility of both PET and MRI data sets must be demonstrated, thus indicating that the phantom chosen shows potential as a test object for simultaneous PET/MRI acquisitions. Mathematical and computational/software phantoms are not considered in this review.

**Fig. 1:**
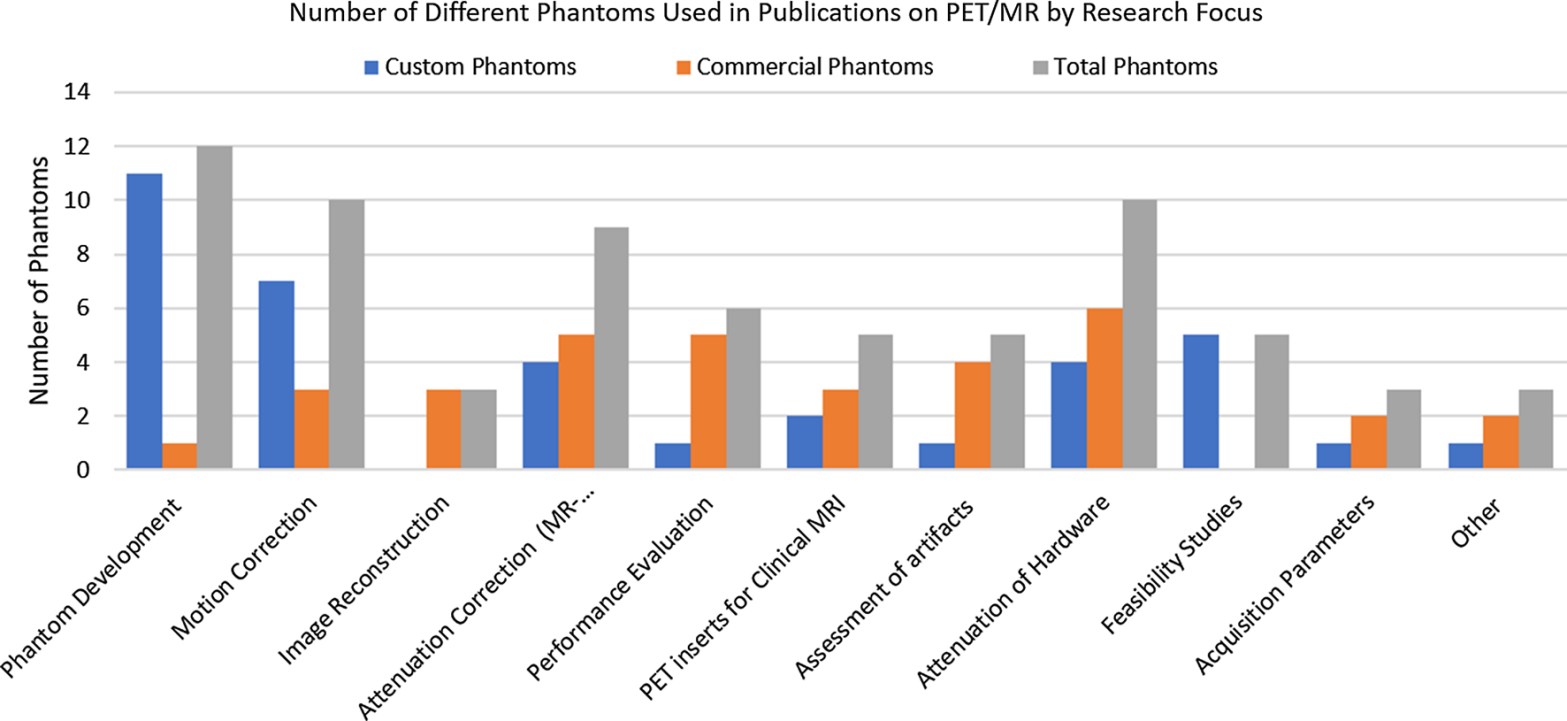
Research and publication categories within MRI identified in this review and the number of unique phantoms used in each category, separated by custom and commercially available phantom designs

The main part of this review begins by summarising the materials used in phantom design and the challenges faced when choosing materials to create PET/MRI phantoms. We then categorise the phantoms identified from literature into two broad categories. First, we cover geometric phantoms, those that feature simple geometries such as those designed for quality assurance programs, which may be commercially available or manufactured in-house. The second is anthropomorphic phantoms, designed to replicate specific human anatomy, physiology or tissue properties in PET/MRI for which there are commercially available and in-house manufactured solutions. For each category we present phantom designs and the use cases demonstrated in recent publications, a summary of which can be seen in Fig. 1. We then discuss how the presented phantoms address key research areas posed in the field of PET/MRI, and the developments still needed to create widely available, reproducible phantoms suitable for simultaneous PET/MRI acquisitions.

## Materials in phantom design

Ensuring the materials chosen for phantom development exhibit the properties required for both PET and MRI imaging simultaneously can be challenging as PET/CT phantoms focus on radiotracer distribution and electron density of materials, whilst MRI phantoms are optimised to the desired proton relaxation times. A list of the polymers mentioned in this review and their abbreviations are shown in Table 1. Polymethyl methacrylate (PMMA) is the preferred choice in commercially available phantoms as it is strong, transparent and offers a similar X-ray radiation attenuation to human tissue [1]. However, it is not MRI visible, so the attenuation properties of PMMA phantoms cannot be correctly derived in PET/MRI acquisitions and so alternative materials for phantom development have been explored.

**Table 1:** Abbreviations and corresponding full names of polymers used in the manufacture of phantoms featured in this review

Abbreviation	Polymer name
PA	Polyamide
PE	Polyethylene
PEEK	Polyether ether ketone
PMMA	Poly(methyl methacrylate)
PP	Polypropylene
PTFE	Polytetrafluoroethylene
PU	Polyurethane
PVA	Polyvinyl alcohol
PVC	Polyvinyl chloride

Phantom development, including characterisation of the materials, is an active area of research among groups performing phantom studies in PET/MRI, with 12 phantoms featured in publications on phantom development between 2011 and 2021, as shown in Fig. 1. In-house manufactured anthropomorphic and tissue equivalent phantoms in particular are often featured in a dedicated publication to describe the phantom design and manufacture, or demonstrate its properties as a suitable PET/MRI phantom.

### Tissue mimicking materials

A recent review on tissue mimicking materials has been published by McGarry et al. [10] providing an overview of the material properties and manufacture methods across a range of imaging modalities. The authors outlined both the requirement for and challenges present in developing tissue mimicking material displaying the desired properties for multiple imaging modalities. Here, an in-depth focus on the materials used as tissue analogues in PET/MRI phantom development is presented, a summary of which is provided in Table 2.

**Table 2:** Materials used in anthropomorphic phantoms categorised by the tissues they have been used to represent

Tissue type	Materials used
Brain	Agarose gel, PE, saline soaked cotton, water
Bone	Gypsum plaster, dipotassium phosphate, cadaver, petroleum jelly
Soft tissue	Agarose gel, methyl-cellulose gel, gelatin, gel (unspecified), saline, water
Adipose	Peanut oil, silicone
Tumour/lesion	Wax, agarose gel, gel (unspecified), gelatin, plastic or glass spheres
Heart	Silicone, gel (unspecified), water
Lung	Rubber balloon, cadaver, silicone
Other tissue	Rubber balloon, hosepipe, silicone, agarose gel
Other materials	PMMA, VeroClear, Agilus 30 clear, PU, PU/PVA mix

Three phantoms were developed to include animal cadavers of porcine and bovine origin for bone and lung tissue. Animal cadavers can provide materials with similar structure and properties to the equivalent human tissues; however, these may be altered in ex vivo samples [11]. Two of these phantoms were used as part of the validation process for MRI-based attenuation correction [12, 13]. One study described a phantom built with animal femur bone and lung lobe as a feasible solution to create tissue equivalent phantoms for PET, CT and MRI [14]. However, consideration is required as to how components will be cleaned between experiments, to ensure a like-for-like replacement for cadavers at appropriate time intervals and to accommodate for the embalming process. Whilst there are benefits to utilising such phantoms in work around tissue classification in MRI-based attenuation techniques, particularly where a range of tissue types are represented [15], there are no known and verified relaxation properties or attenuation coefficients to validate against. Applications are also limited by a lack of anthropomorphism and the limitation of introducing a meaningful and realistic radiotracer distribution to a cadaver.

#### Soft tissue

Three approaches to modelling brain tissue are encountered in this review. The Hoffman phantom [16] (Data Spectrum Inc.) and Iida phantom [17] both form models of the human head using polymers for white matter and an open compartment filled with radiotracer solution for grey matter. This provides an ideal radiotracer distribution, but the polymer structure is invisible to MRI. Saline soaked cotton as used by Okazawa et al. offers limited use as a brain tissue surrogate given that in the form presented no radiotracer is administered [18]. Agarose gel used by Harries et al. benefits from displaying closer MRI signal properties to soft tissue than water or saline [19]; however, establishing a detailed grey and white matter structure could be challenging in terms of structural integrity, level of detail achievable and how a heterogeneous radiotracer distribution could be established or even reproduced.

Agarose, gelatin and methyl-cellulose gels are used more widely as soft tissue surrogates in several phantoms identified for this review [13, 14, 20–23]. The ability to customise the MRI relaxation properties of gels with relative ease at manufacture by varying the concentration of gelling and contrast agents, demonstrated extensively by Gillmann et al. [21], allows for flexibility in the number of tissue types represented. Gels can be used to fill cavities or moulded to hold structure without a casing, both of which provided a reproducible geometry. Moulded structures such as tumours or lesions may then be placed inside a larger gel tissue surrogate [23]. In phantoms simulating cardiac and respiratory motion [20, 24–26], gels offer an alternative to water whilst maintaining the flexibility to allow movement to occur. Additionally, gels are easily manufactured in-house without the need for specialist equipment.

However, if using short half-life radiotracers such as [$^{18}$F]FDG, gel tissue surrogates must be remade each day they are required, so manufacture and setting time must be accounted for to ensure sufficient radiation is detectable at time of scanning. Alternatively, a long half-life radionuclide could be suspended within the gel, but this would require careful consideration of the safe long-term storage and disposal of the radioactive gel. Furthermore, each manufacture session is subject to a level uncertainty. McIlvain et al. [27] have encouraged sites to understand the impact the variations in manufacture may have on the material properties. Using gels inside intricate casings may introduce air bubbles during manufacture, and be difficult to clean for reuse. Finally, all of the phantoms identified in this review use homogeneous radiotracer distribution throughout each gel tissue surrogate; however, this does not represent every clinical scenario as highlighted by Valladares et al. [28], who presented proposed solutions for heterogeneous tissue equivalent materials for medical imaging phantoms.

#### Bone

Bone material analogues are challenging to create for PET/MRI applications as the chosen material should exhibit a cortical bone, or average bone, electron density for realistic PET attenuation, whilst maintaining very short T1 and T2 relaxation times.

Phantoms with bone surrogates were most commonly created in house using gypsum plaster [19, 21, 29] doped with iodine CT contrast agents and either gadolinium MRI contrast agents or copper sulphate to modify the linear attenuation coefficient and relaxation times respectively. Chandramohan et al. [29] assessed the radiological properties of several samples of gypsum plaster mixed with varying concentrations of each doping agent for comparison with human bone. They found that plaster doped with copper sulphate provided the combined radiological properties suitable to mimic cortical bone; however, the relaxation properties of the material were unstable over time and were affected by the introduction of microbubbles into the plaster during manufacture, warranting further investigation. Harries et al. [19] casted a skull from iodine doped plaster, which is classified as bone in five out of six MRI-based attenuation maps, but results in an underestimation of PET activity within the phantom of on average 5%, to a maximum of 11%. Gillmann et al. [21] took additional steps to create bone structures that also include a bone marrow surrogate of petroleum jelly mixed with dipotassium hydroxide, allowing for lesions to be place inside. As the use of PET/MRI in areas of the body with larger bone structures increases, the differentiation between cortical bone and other features such as bone marrow may become more relevant in phantom experiments. Dipotassium phosphate was used as a bone equivalent material in the Iida phantom [17]; however, as a liquid solution it is highly visible in MRI images and so is not classified as bone in MRI-based attenuation maps [30].

Overall, gypsum plaster has presented a promising solution to replicating the material properties of cortical bone in PET/MRI phantoms and is a widely available crafting material. However, further work is required to assess the effects of different manufacturing methods and the long term stability of the material properties of doped plaster, as has previously been performed for agarose gels in MRI phantom work [27].

#### Adipose tissue

Fats are largely ignored across the phantoms produced, with only individual use of silicone [19] and peanut oil [21]. Of these materials, peanut oil provided MRI relaxation properties close to those of adipose tissue, whilst silicone exhibits a much shorter T2* [19]. Phantoms designed to represent anatomy such as the breast would benefit from further investigation into use of materials with radiological properties similar to adipose tissue.

### Polymers and 3D printing

Casings for organs and the overall phantom were manufactured using a variety of polymers. Most commonly, PMMA and PU were used, but 3D printable polymers were also used for individual organs. Of the phantoms featured in this review, the Iida phantom [17] provided attenuation properties for the 3D printed polymer and Talalwa et al. [31] demonstrated the dielectric properties of their proposed porous MRI-visible 3D printed polymer made of a PU/PVA mix. Gillman et al. [21] reported the CT Hounsfield units and MRI relaxation times for VeroClear (Stratasys) but this is not referenced to any tissue value, suggesting it was not selected to exhibit tissue equivalent properties.

In their systematic review to identify trends in the use of 3D printing in the development of medical imaging phantoms, Fillipou and Tsoumpas [32] found that radiological properties are not commonly tested by manufacturers for 3D printing. However, Rausch et al. [33] have recently created a phantom using 3D printed polymer RGD252 (Stratasys), previously demonstrated as MRI visible [34], that is visible in both modalities for simultaneous PET/MRI acquisitions.

NIST have released two publications [35, 36] demonstrating CT and MRI properties for a range of commercially available 3D printable polymers for comparison with human tissue. Although several challenges exist to utilising 3D printed phantoms in multimodal imaging [32], future work would benefit from further consideration of the MRI relaxation and PET attenuation properties when choosing polymers from which to 3D print PET/MRI phantoms. In particular, this would encourage the correct classification of structures for MRI-based attenuation correction. Silicone and rubber balloons used to represent the heart [20, 24], lung [25, 26] or bladder [21] also lack MRI visibility. Whilst this is less of an issue for lung tissue as this is typically classified as air, care must be taken to ensure this does not lead to an under estimation of PET activity.

### Summary of materials in PET/MRI phantom design

Gel-based phantoms with radiotracer introduced prior to setting provide the option of creating phantoms with soft tissue equivalent MRI relaxation times with a uniform radiotracer distribution; however, radiotracer solutions of water or saline are also commonly used and may be doped with gadolinum-based contrast agent. Gypsum plaster has been the most utilised option for bone analogue materials; however, further work is required to establish the stability of the phantoms produced and reproducibility of manufacture methods. Few options have been explored in mimicking adipose tissues. Advances in 3D printing and the range of polymers available may offer solutions in the future in creating phantoms for simultaneous PET/MRI, particularly in light of the work performed in assessing the radiological properties of materials available. However, these materials generally lack the large scale manufacture and shelf life capable of creating reproducible phantoms able to be distributed across multiple sites. Subsequently, established polymers in phantom design such as PMMA continue to be used widely for building PET/MRI phantoms despite their lack of signal in MRI.

## Geometric and homogeneous phantoms

**Fig. 2:**
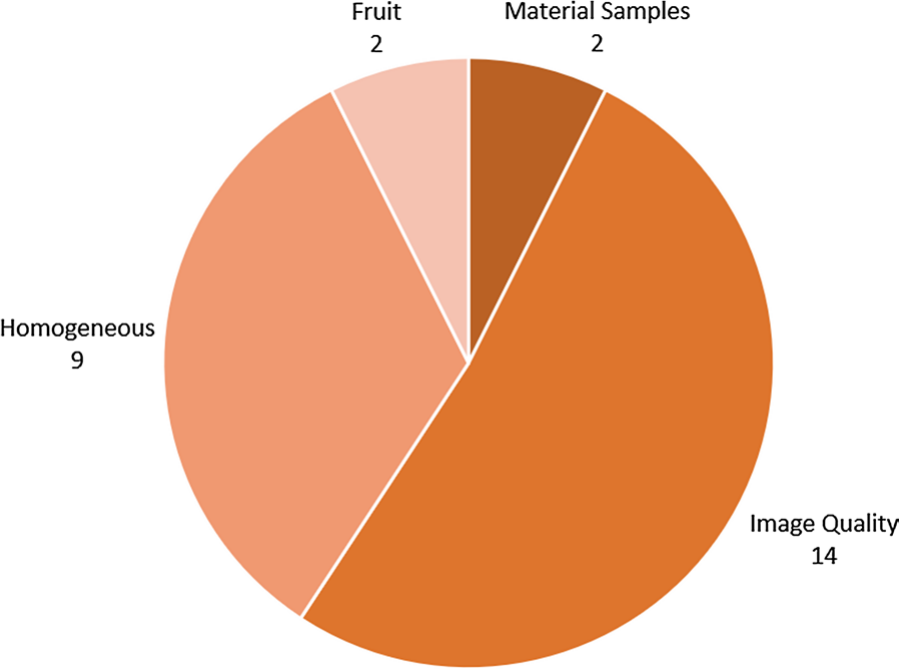
Physical PET/MRI phantoms categorised by design features. Material samples consist of small amounts of material for characterisation. The fruit category refers to the use of modified fruits as PET/MRI test objects, although these are not discussed in detail

Geometric phantoms are those which feature simple geometric inserts and cannot be considered anthropomorphic. Homogeneous phantoms are phantoms for which there are no inserts, providing a uniform fluid distribution within the phantom body. Twenty-five geometric or homogeneous phantoms were identified in this review, a breakdown of which is shown in Fig. 2. Although this is only slightly more than half of the phantoms encountered, they appear in 59 of the 92 publications reviewed. Thirteen of these take the form of geometric phantoms, often used in quality assurance measurements. The 9 homogeneous phantoms take the form of a cylindrical container, bottle or canister filled with a single fluid, and are considered to be commercially available.

Of the geometric phantoms, 9 are custom designed and included in 14 publications. 31 publications use 5 commercially available geometric phantoms, the most common being the International Electrotechnical Commission (IEC) NEMA Body Phantom featured in 19 publications. The predominant use cases for these phantoms within PET/MRI literature are measuring image quality and scanner performance, verifying MRI-based attenuation correction and image reconstruction methods and generating attenuation maps for radio-frequency coils or other hardware.

### Homogeneous phantoms

**Fig. 3:**
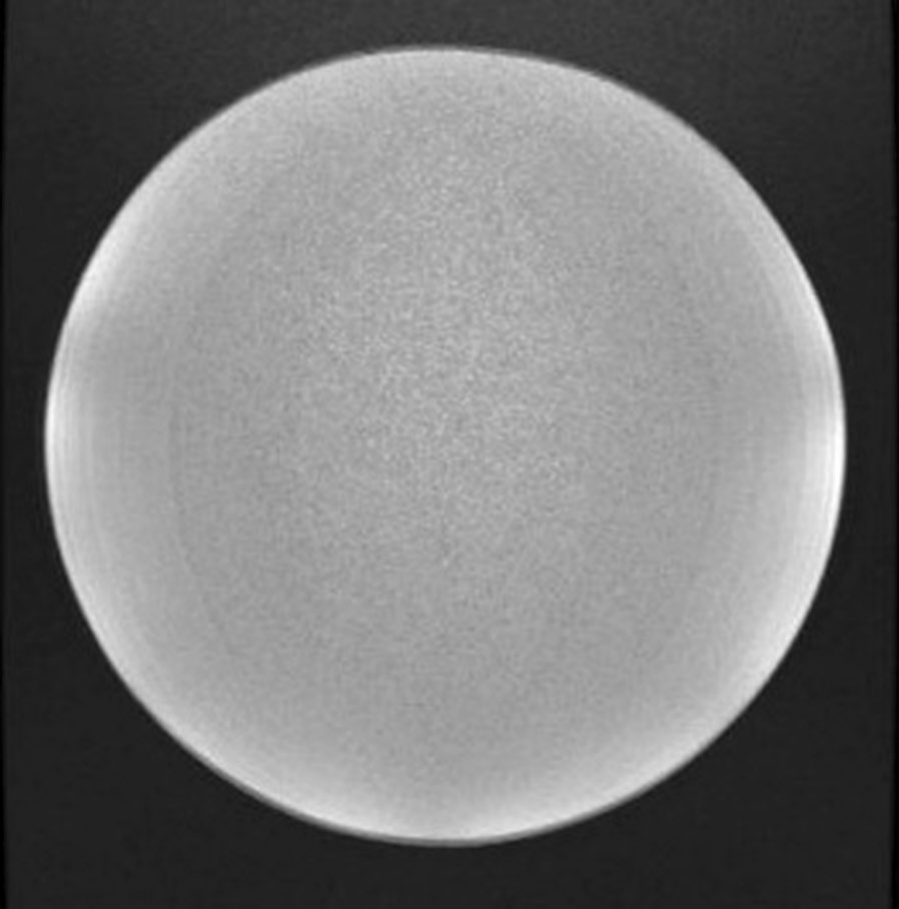
Cross section of a uniform phantom acquired in MRI (GE Signa PET/MRI scanner)

Homogeneous phantoms act as a test object filled with a single, homogeneous fluid, an example of an MRI acquisition for which is shown in Fig. 3. These are simple to use and give indication of performance through assessment of image uniformity and image noise. They are easily accessible, given that they can be fashioned from any water-tight, MRI safe container. The variety of containers available allows researchers to tailor the size of the phantom appropriately.

MRI signal properties can be improved by using nickel sulphate [37], sodium chloride [38] or introducing a gadolinium-based contrast agent [39] alongside the radioactive tracer to reduce artifact in large phantoms. This allows researchers to create solutions that can be imaged with both PET and MRI; however, containers are not MRI visible and so cannot be included in MRI-based attenuation maps. This can be mitigated by using containers with thin walls to minimise PET attenuation by the container and allow for attenuation maps to be approximated to the fluid volume. No two groups of authors used the same uniform phantom, and phantom volumes range from 160 mL to 29 L. This reflects the differences in studies presented, but also suggests a lack of a standardised approach to assessing uniformity in PET/MRI.

### Commercially available geometric phantoms

All commercially available geometric phantoms featured in this review are phantoms initially designed for PET or Nuclear Medicine use and licensed by Data Spectrum Corporation:

- IEC (NEMA) Body Phantom
- Esser (ACR) PET Phantom
- Jaszczak Phantom
- NEMA 94 PET Phantom
- Mini Deluxe Phantom

They are widely available and many sites with existing Nuclear Medicine and PET facilities are likely to already possess a subset of these phantoms in order to adhere to quality assurance guidelines [8]. In particular, the IEC (NEMA) Body Phantom continues to be recommended for PET and PET/MRI acceptance testing and quality assurance under the NEMA NU-2 (2018) standard [7, 8]. It consists of a large, elliptical PMMA container with a central cylinder filled with polystyrene, around which hollow spheres are suspended from one end of the phantom. The standardised geometry, manufacture and protocols used ensure comparable measurements between sites and promote reproducibility across different scanners. This is reflected in literature through publications where the IEC Body Phantom was used to evaluate PET/MRI scanner performance [40–42] and compared performance and protocols to PET/CT [43–46] prior to introducing patients studies. Krokos et al. [41] illustrated the crucial example of ensuring standardisation between several PET/MRI scanners for use in multicentre dementia trials.

A significant drawback of these phantoms is the inability to create accurate MRI-based attenuation maps due to the widespread use of PMMA for the phantom body and its lack of visibility in MRI. Ziegler et al. [47] compare results generated from the NEMA NU-2 Protocol for a Siemens Biograph mMR using images reconstructed with both an MRI-derived attenuation map and a CT-derived attenuation map. They found that using an MRI-derived attenuation map decreased contrast recovery in radioactive spheres, increased contrast recovery in non-radioactive spheres and increased background variability, indicating a degradation in both image quality and PET quantification due to insufficient correction for attenuation. Their recommended solution was to acquire a CT scan of the phantom separately in order to generate a suitable attenuation map that can be registered to the MRI or PET images to perform image reconstruction of the phantom offline. Further comparisons [48] extended this to include the MRI-based attenuation correction methods employed in the Philips Ingenuity TOF and GE Signa PET/MRI scanners for phantoms, as well as assessment of clinical MRI-based attenuation correction in phantom studies. Replacement of the MRI-based attenuation map with a registered CT-based map appears to be the preferred solution in publications included in this review where geometric phantoms are used. As such, vendors provide a predefined PET attenuation map in the scanner reconstruction software for the IEC Body Phantom for PET performance testing under the NEMA protocol; however, this doesn’t extend to all commercially available phantoms.

#### Addressing MRI visibility of commercially available phantoms

In an earlier publication, Ziegler et al. [49] assessed a variety of fluid fillings to improve MRI visibility and attenuation map generation for the IEC Body Phantom. By varying the fluid filling for the phantom, they were able to significantly improve homogeneity in the MRI images and reduce bias in PET data resulting from inconsistencies in the MRI-based attenuation map. The key finding was that a pure water radiotracer solution as recommended in the NEMA NU-2 protocol was the least suitable of the fluids assessed, with triethylene glycol providing the greatest homogeneity. However, none of the fluids presented appear to provide a robust solution for routine use due to risk of toxicity, additional cleaning requirements and costs. Use of any alternative fluids also does not address a lack of phantom housing visibility in MRI.

Currently, no geometric phantoms for the quality assurance of PET/MRI systems exist on the market that are both PET and MRI visible. Whilst many performance issues will be detected by separate testing of MRI and PET components of the scanner, enabling quality assurance phantoms to undergo simultaneous imaging and use the same attenuation correction and image reconstruction methods as would be used clinically is highly desirable. This would act to both confirm the performance of these systems and to allow phantom testing to form part of wider imaging protocol validation projects. Additionally, there is little standardisation in MRI quality assurance programs [7], increasing the likelihood of cross-site variation in PET quantification given clinical reliance on MRI-based corrections. It is clear that more work is to be done in this area, either through alteration of MRI sequences used to generate phantom attenuation maps, or through the development of phantoms from materials exhibiting properties that allow for their visualisation in MRI acquisitions.

### Custom designed geometric phantoms

The custom designs of geometric phantoms featured in this review are all borne from a requirement to test features for which no commercial option existed, often extending beyond performance testing of clinical PET/MRI scanners.

#### Rectangular whole body phantom

Braun et al. [50] assessed the feasibility and performance of continuous table motion acquisitions in PET/MRI by developing a large polypropylene (PP) rectangular phantom to approximate the dimensions of the human body, which was separated into cubic compartments. Each compartment had holes of varying size drilled into the sides to visually assess image quality and quantitatively assess resolution in both PET and MRI. The phantom was filled with a radiotracer solution. Whilst this phantom addressed the question it was designed for and was able to assess both PET and MRI performance [50, 51], it still suffered the same set-backs as commercially available phantoms and requires an external CT-based attenuation map to be used during PET reconstruction.

#### Phantoms to study motion correction

In their assessment of motion correction methods, cylindrical acrylic phantoms filled with water and containing inserts featuring $^{22}$Na point sources were used by two groups [52, 53]. Given that a CT-based attenuation map was required for the reconstruction of PET data in order to account for the trolleys and bases used in these studies, the ability to generate an MRI-based attenuation map may have not been a design priority. However, in both cases motion correction was informed by MRI data and so MRI visibility of the phantom was crucial. Previously, a phantom made of PVA cryogel with radiotracer introduced during manufacture was demonstrated as a PET and MRI visible phantom able to undergo non-rigid movement [54].

#### MRI visible polymers in phantom design

Rausch et al. [33] demonstrated the first PET/MRI phantom in-house-made with MRI visible housing. The cylindrical phantom had rod features on the bottom lid, whilst keeping the top section of the phantom homogenous. The phantom was filled with an aqueous solution of [$^{18}$F]FDG, sodium chloride and a gadolinium-based MRI contrast agent. They compared PET reconstructions of the phantom performed using no attenuation map, attenuation maps generated from the Siemens Biograph mMR DIXON sequences, an optimised phantom-specific MRI-based attenuation map and PET/CT images. Their work demonstrated that polymers are available for phantom construction that can be imaged by simultaneous PET/MRI, although the MRI-based attenuation map overestimated the phantom extent. Further work should be done to verify approaches for optimising MRI-based attenuation maps for phantoms using MRI visible polymers across different scanner models.

#### Geometric phantoms for brain PET/MRI

Grant et al. [55] first demonstrated a 3D printed geometric image quality phantom for performance testing the BrainPET (Siemens Healthcare) [56] MRI insert, for which the full characterisation is presented by Bieniosek et al. [57]. It was a cylindrical phantom with one empty quadrant containing plastic rods and three quadrants of solid polymer with cylindrical holes in a range of diameters. The empty spaces were then filled with a radiotracer solution. The group used the phantom for two further publications [58, 59]. The use of 3D printing in phantom development is increasing due to the widespread availability of 3D printers and low manufacturing costs [32], but the field lacks consistent assessment of the reproducibility in 3D printed phantoms. The publication of the phantoms design, manufacture and characteristics [57] facilitates the ability for other groups with 3D printing capabilities to produce and use this phantom in further work. Additionally, they validated the manufacture method through replication of a commercially available phantom and compared PET/CT acquisitions of the commercial and in-house printed phantoms [57], confirming its suitability for phantom manufacture. However, the phantom design does not address MRI visibility of the phantom housing and attenuation correction adequately.

Size appears to have been the driving factor for custom phantoms in brain PET. A cylindrical PMMA phantom with hollow rods and a polytetrafluoroethylene (PTFE) insert was utilised to validate the use of an orbiting transmission point source for attenuation correction of PET data compared to that of an MRI-based attenuation map [60]. The authors addressed the challenges in generating accurate MRI-based attenuation maps through implementation of a transmission source mechanism in generating attenuation maps for brain PET/MRI [60]. However, this was then used to replace the MRI-based attenuation map entirely with one derived from the transmission source measurements, requiring the use of a specialised MRI coil that is applicable to head imaging only.

#### Geometric phantoms in radiosurgery planning

Lim et al. [61] used two acrylic phantom designs in their study on the feasibility of using [$^{11}$C]methionine PET in Gamma-Knife radiosurgery planning. This required phantoms that are suitable in validating multiple imaging modalities as they are introduced into the radiotherapy planning framework. These phantoms were imaged with CT, MRI and PET to assess geometric accuracy, image registration and quantitative PET within the Gamma-Knife radiosurgery planning system.

#### Summary of geometric phantom designs

Each of these phantoms is used at one site and designed to address specific research questions, with only one fully presented and characterised to promote its use by other institutions. Characterisation of materials and methods presents a barrier to widespread adoption of in-house manufacture methods such as 3D printing [32], as each group must then perform these measurements. The design and verification of a phantom require a significant time commitment, and so distribution of this information is valuable to the imaging community to accelerate phantom development. Most recently, an MRI visible polymer has been demonstrated as suitable for PET/MRI use [33], providing a potential solution to MRI-based attenuation correction issues in phantom studies.

## Anthropomorphic phantoms

**Fig. 4:**
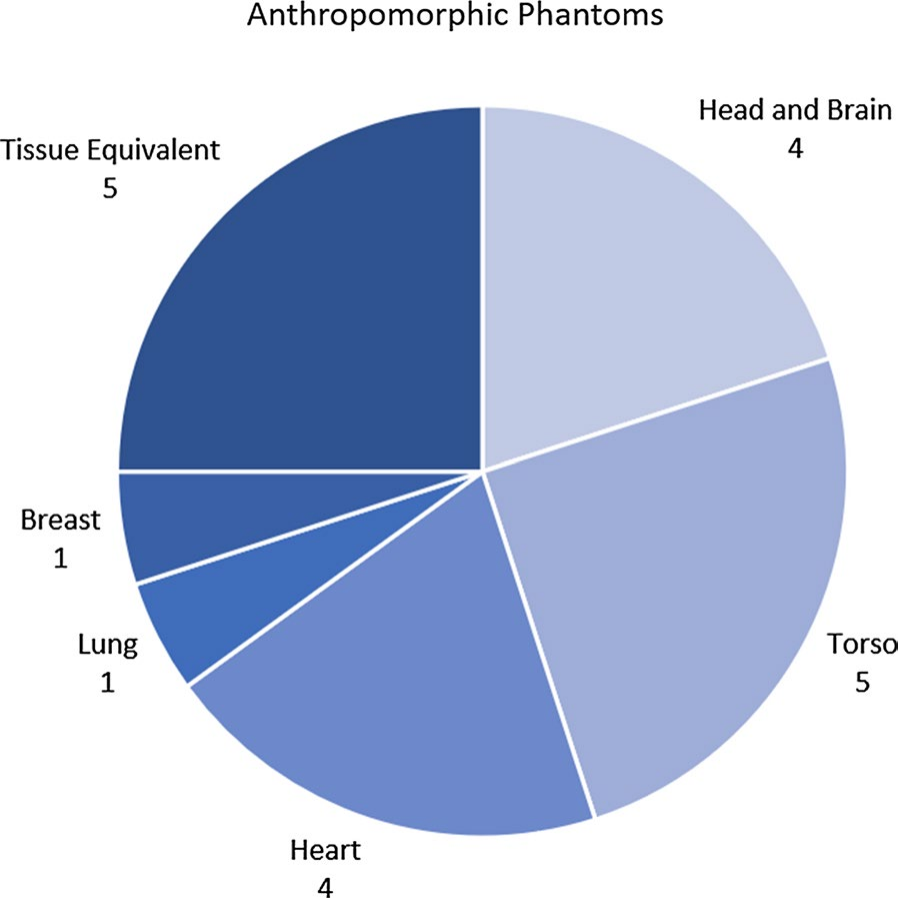
Anthropomorphic PET/MRI phantoms categorised by the area of human anatomy they represent

A total of 20 anthropomorphic and tissue equivalent phantoms were identified in this review, of which 16 are custom-designed or in-house-manufactured. These phantoms cover 5 areas of human anatomy, as displayed in Fig. 4, with a final category describing phantoms that contain materials equivalent to specific human tissues but do not represent any specific anatomy.

Head and brain phantoms are the most published category of anatomical phantoms at 16 publications. This reflects the commonly desired clinical PET/MRI application of neurology and the subsequent areas of research addressing attenuation correction in brain imaging [62]. Correcting for bulk and physiological motion are also an important area of interest, the mechanisms of which are beyond the scope of this review, but are presented by Polycarpou et al. in their recent review [63]. Physiological motion in particular requires the use of phantoms able to replicate the anatomical geometries and motion required. Again, this is reflected in the phantoms designed and their publications with 7 torso and cardiac phantoms used across 14 publications.

### Commercially available anthropomorphic phantoms

As with geometric phantoms, the commercially available options utilised for PET/MRI are phantoms originally designed for Nuclear Medicine and PET. These phantoms are well established within the field due to the necessity of administering a radioactive tracer for PET and $\gamma$-camera imaging limiting the recruitment of volunteers for investigative work. By comparison, MRI phantoms often focus on quantitative performance through simple geometries [64], although some anthropomorphic MRI phantoms are commercially available and tissue equivalent materials are in use throughout the MRI community [6].

#### Hoffman phantom

The Hoffman brain phantom [16] (Data Spectrum Corporation) is one of the most popular anthropomorphic phantom featured in 7 publications identified by this review. It is a PMMA cylinder containing 19 composite polycarbonate plate inserts which, when filled with fluid, simulate normal [$^{18}$F]FDG radiotracer distribution in the brain and a realistic MRI proton density distribution. Optional inserts are also available to simulate defects. The Hoffman phantom has been used to assess the performance of PET inserts in clinical MRI scanners [65–67], evaluate motion correction methods [68, 69], the demonstrate of PET image reconstruction methods [70] and MRI-based attenuation correction [71] methods. This is the only commercially available phantom identified within this review that is also advertised for MRI use, although the polycarbonate plates are not visible in MRI. The design lacks a skull component for a realistic attenuation profile, it does not replicate brain tissue relaxation properties in MRI, and the cylindrical design does not conform to human head geometry, which are major limitations for its use as a human head surrogate in PET/MRI.

#### Torso phantoms

Two commercially available phantoms of the Thorax were used: the elliptical Lung-Spine [72] and Anthropomorphic Torso Phantom [73]. Both phantoms are licensed by Data Spectrum Corporation and are constructed from PMMA with polystyrene-filled lung inserts, solid PTFE spine insert and an optional PMMA cardiac insert modelled on the left ventricle and myocardium. The Anthropomorphic Torso phantom also features a liver insert. The phantom body, cardiac and liver inserts are then filled with radiotracer solution. A PU cardiac insert (Radiology Support Services) representing two cardiac chambers and the myocardium was used independently in one study [74].

#### Commercially available anthropomorphic phantom summary

All of the commercially available anthropomorphic phantoms are constructed from PMMA or PU, and so any solid material is not MRI visible. Despite the concerns raised around MRI-based attenuation correction in geometric phantoms, many studies use MRI-based attenuation maps to reconstruct PET data for these anthropomorphic phantoms, including two studies actively using these phantoms to assess the impact of using MRI-based attenuation correction [71, 73]. This may be because the differences between MRI-based and CT-based attenuation correction in PET quantification still need to be quantified prior to clinical use, or because MRI-based attenuation methods based on tissue segmentation from anatomical maps [75] are more easily applied to anthropomorphic phantoms than non-anatomical shapes.

The current commercially available phantoms are only able to assess brain and cardiac protocols. This excludes some key applications of interest in clinical PET/MRI, such as [$^{68}$Ga]-PSMA imaging in prostate cancer patients [76]. Crucially, no commercially available anthropomorphic phantoms are able to accurately represent human tissue in both PET and MRI.

### Custom anthropomorphic phantoms

Whilst an accurate representation of anatomical geometry is a key aspect of design across all anthropomorphic phantoms, many in-house manufactured phantoms identified in this review take additional steps to achieve both PET and MRI tissue equivalence, or mimic additional physiological properties.

#### Head and brain phantoms

Three human head phantoms were developed between 2011 and 2021. The first of these was the Iida Brain phantom [17], initially developed for use in PET and single-photon emission computed tomography (SPECT) imaging, it is designed to represent a realistic human head contour and include a cavity around the brain material filled with dipotassium phosphate to simulate the skull. Both of these features address some of the limitations presented by the Hoffman phantom in brain imaging. The phantom was manufactured through 3D printing, for which Iida et al. [17] demonstrated a high degree of reproducibility. The Iida phantom has been assessed for PET/MRI use, with Johansson et al. [77] providing a description of potential applications, highlighting the issues around a lack of white matter radiotracer distribution and difficulties with MRI-based attenuation correction when scanned with the Phillips Ingenuity PET/MRI scanner, but ultimately concluding it to be a useful test object. The Turku PET Centre group later used the phantom in three additional PET/MRI publications [30, 71, 78] including an international multi-centre study.

Harries et al. [19] presented their solution for a human head phantom with a focus on mimicking both MRI and PET tissue properties. The authors detailed the relevant properties of their chosen materials as well as the limitations in the design and manufacture of this phantom. In particular, the design did not mimic the structures of grey and white matter in the brain, so whilst the feasibility of manufacturing such a phantom and its compatibility with MRI-based attenuation methods was demonstrated, applications may be limited due to the reduced detail in brain structure compared to the Iida and Hoffman brain phantoms. Additionally, this phantom necessitates in-house manufacture due to the use of short shelf-life materials such as agar. As a result, a centre reproducing this phantom should still perform validation measurements to ensure the desired material properties and geometries have been achieved. Finally, Okazawa et al. [18] used a human skull filled with saline-soaked cotton and radiotracer filled rubber tubes to represent arteries. This phantom was specifically designed to address the authors research question regarding the reliability of quantitative [$^{15}$O] imaging in the carotid arteries using PET/MRI.

#### Torso phantoms

Larger phantoms representing the pelvis [21] [38] and torso [22] have also been designed and used within PET/MRI. These phantoms feature multiple organs with differing tissue properties and radiotracer uptake, and so 3D printing was utilised to enable the individual design of each component. Currently, the printable materials commonly available to sites without an extensive manufacturing department are limited in the tissue-equivalent properties they can display [10, 32]. As a result, groups developing these phantoms often opted to either 3D print an outer shell of the organ of interest which may be filled with an appropriate tissue-equivalent material, or use a 3D printed mould to cast a model using a gel or wax.

The ADAM PETer pelvis phantom [21] was designed for applications regarding the use of multimodal imaging in radiotherapy. It was based on the ADAM-pelvis phantom [79], by further developing the model to be compatible with PET imaging in addition to CT and MRI with the aim to utilise the phantom in the optimisation of PET/MRI guided radiotherapy for prostate cancer patients [21]. The ADAM PETer also introduced 3D printing into the phantoms manufacture to improve the modular design. The phantom featured a large number of materials to simulate various tissues, which were extensively described along with the phantoms lengthy assembly process. However, the authors reported that due to a modular design, switching out of organs of interest was less laborious once the phantom was constructed and so allowed customisation of the phantom for several clinical scenarios [21]. Further work validating the reproducibility of 3D printing organ modules for this phantom would facilitate its use external to the home institution.

Another pelvis phantom [38] consisting of a PMMA container filled with a radiotracer solution has also been tested. It was noted that the phantom is described in other works excluded by the review criteria [80, 81] as featuring multiple organ inserts; however, these features were not highlighted in any of the PET/MRI work.

**Fig. 5:**
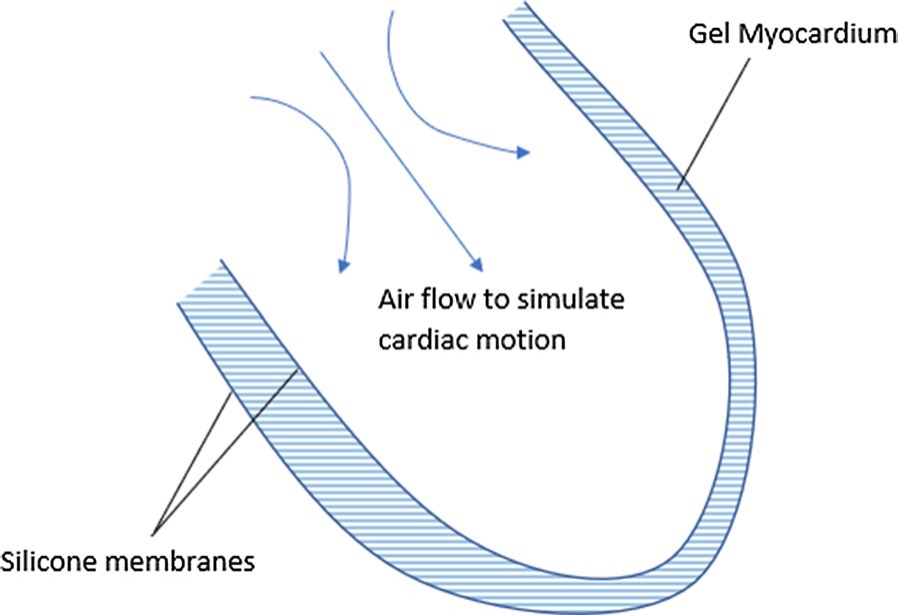
A schematic demonstrating the general design concepts of phantoms to simulate cardiac motion, as used by [20, 22, 24]

The Wilhelm Anthropomorphic Torso Phantom [22] has been developed by the European Institute of Molecular Imaging as a phantom for PET, CT and MRI capable of replicating cardiac and lung motion. Bolwin et al. [22] described the design, manufacture and characterisation extensively; however, this phantom has been used by the institute in several works addressing motion correction in PET/MRI since 2011 [82–85]. Wilhelm features inflatable silicone lung inserts and a silicone cardiac insert. Both sets of inserts are piston driven to induce motion. A double silicone membrane is used for the cardiac insert to create an outer layer that can be filled to represent the myocardium, a schematic for which is shown in Fig. 5. Lung inflation is controlled from the base of the lungs by a rubber membrane to simulate the diaphragm. A liver compartment is also included. The phantom could be customised by the choice of fluid filling for each compartment and additional wax spheres mounted within the phantom as lesions. Models of the heart and lungs were 3D printed to create moulds on which to shape the silicone. No skeletal structure was featured in the phantoms’ design which may limit its application in other areas of interest such as MRI-based attenuation correction.

Motion correction is an on-going area of research with different methods appropriate to assess different types of motion [63]. As such, the Wilhelm phantom occupies a niche the needs of which have not been met by commercial vendors. However, this phantom is highly complex, having been developed over many years [22], with a multitude of materials and manufacture methods required for its construction.

#### Cardiac and lung phantoms

Inflatable double-membrane cardiac phantoms of a reduced complexity have also been demonstrated as suitable for motion correction applications in PET/MRI [20, 24]. These phantoms focussed exclusively on cardiac motion, placing one balloon inside another, filling the interspace with a mix of radiotracer, gadolinium-based contrast agents and gelling agent to represent the myocardium. This was then suspended within a second radiotracer gel mix to simulate a soft tissue background. Similarly, a single membrane balloon has been used to simulate respiratory motion [25, 26]. The materials in these phantoms are more widely accessible to sites without the large time, cost and facilities to create a phantom as complex as the Wilhelm phantom. However, these phantoms were not described as extensively and are less representative of the clinical scenario both anatomically and in terms of physiological motion.

In myocardial perfusion, O’Doherty et al. [86] used a different phantom, initially designed and described as an MRI phantom [87], to demonstrate the feasibility of first pass myocardial perfusion imaging in PET/MRI. The phantom featured four chambers simulating the atria and ventricles of the heart, with two cylindrical compartments each side representing the myocardium. Tubes representing veins and arteries have been arranged such that the flow of fluid through the phantom simulates entry to the heart from the vena cava. Water is delivered to the phantom from an external pump and control unit. The phantom was demonstrated as suitable for PET/MRI; however, the lack of tissue equivalent materials and true anthropomorphism may impact how applicable any results are to clinical imaging. Matusiak [88] presented a multimodal heart phantom (MHP) constructed of PMMA featuring two chambers to simulate left and right ventricles and a surrounding space to simulate the myocardium. The imaging compatibility of the phantom across SPECT, PET, CT and MRI was demonstrated through visual assessment and image registration between the imaging modalities [88], but no quantitative data were presented.

#### Breast phantom

A modular breast phantom was presented by Aklan et al. [89]. Breast imaging in MRI uses specialised RF coils for which anthropomorphic phantoms are not typically available. The modular breast phantom consisted of two PMMA domes with optional MRI and PET/MRI inserts. The PET/MRI insert features four sizes of glass sphere to be filled with radiotracer solution assembled onto a cross structure that is MRI visible [89]. This phantom was more of a hybrid between the anthropomorphic and geometric phantoms, as the insert features are more representative of those expected in a quality assurance phantom and when filled with water does not replicate the tissue composition of the breast. However, the design has been well described and offers a solution to performance testing and validation of equipment and protocols for breast imaging in PET/MRI, for which the only alternative demonstrated has been uniform bottle phantoms [39].

#### Summary of anthropomorphic phantom designs

The in-house manufacture of anthropomorphic phantom featured in this review have demonstrated numerous benefits. One is the relatively low cost of producing phantoms where manufacturing techniques such as 3D printing [32] and casting from moulds are used. The other is that increasingly complex phantoms can be developed to address specific needs within the field of medical imaging, as with the Wilhelm phantom [22] and ADAM PETer [21]. The ability to customise these phantoms to simulate multiple clinical scenarios also makes the designs appealing, and may lead to validation for patient specific anatomy as seen developing in other modalities [90]. Use of materials able to display similar PET and MRI properties to human tissues promotes the testing of clinical protocols as would be applied to patients, and by extension greater compatibility with systems such as radiotherapy treatment planning or dosimetry software.

Conversely, the development, manufacture and characterisation of such phantoms can present a significant time commitment and an initial investment cost for sites without existing manufacture facilities. Even well-characterised materials such as agar introduce additional uncertainties to the phantoms properties as they have to be remade for each experiment. Across the literature in this review, description of phantom designs, the material properties assessed, and detail of results presented varies.

Further work is required in this area to increase the utilisation of anthropomorphic phantoms in PET/MRI. The Iida phantom is the only design that appears to have been utilised by multiple institutions [30], but have not demonstrated an adequate level of reproducibility. Significant investment is made into developing and characterising tissue equivalent materials, yet the majority of these designs are housed in a PMMA or PU casing, both of which are not visible in MRI and could affect quantitative PET data when MRI-based attenuation correction is used.

## Discussion

This review has presented an overview of the phantoms used and developed by institutions for PET/MRI during the period 2011–2021. These range from simple containers filled with a uniform fluid to large anthropomorphic phantoms simulating human anatomy and physiology. Commercially available phantoms remain the dominant test objects in performance evaluation of PET/MRI systems, and solutions have been proposed to facilitate their continued use by improving MRI visibility [47, 49].

In-house design and manufacture is a more popular choice for producing anthropomorphic phantoms, as they are often designed with specific clinical scenarios or research questions in mind. Consequently, a wide range of materials, designs and manufacture methods are presented. The characterisation and publication of radiological materials for phantom construction and their comparison to human tissue properties is increasing which, alongside the rise in adoption of 3D printing, facilitates the more widespread use of tissue equivalent materials [32]. Through continued assessment of these properties and the uncertainties in manufacture methods, PET/MRI phantoms can move towards adopting standardised approaches suitable for quantitative imaging assessment and cross-site comparisons.

PET/MRI phantom development would benefit from an increase in options available from the commercial sector and professional bodies pushing for improved solutions for multimodal imaging systems. Of the designs presented in this review, only custom-designed phantoms begin to address the question of creating phantoms exhibiting suitable radiological properties for acquiring quantitative data in both PET and MRI. This restricts not only the dissemination of available phantoms, but also the standardisation of clinical procedures and validation-related software where these phantoms have been used. The impact of this is that patient imaging is then not necessarily comparable across institutions, with particular concern if patients move across different sites during periods of monitoring reliant on quantitative PET/MRI to inform their clinical pathway.

Applying MRI-based attenuation and scatter correction to phantoms remains challenging. No commercially available phantoms exist that exhibit radiological properties suitable for both PET and MRI, either as quality assurance test objects or anthropomorphic phantoms. PMMA remains the preferred choice for constructing phantoms, but is not MRI visible and is often either omitted from or incorrectly classified in MRI-derived attenuation maps. Polymers available for 3D printing may offer some solution to this, with MRI visible options available. Commercial vendors are key stakeholders in PET/MRI phantom development, offering standardised manufacture, material properties and rigorous quality control to their products. As PET/MRI enters routine clinical use, covering a wider variety of applications, it can only be expected that the necessity for studies performed using widely available, reproducible phantoms increases.

## Conclusion

Several commercially available phantoms have been demonstrated as appropriate for limited use in PET/MRI studies, although no vendor has yet released a phantom specifically designed and optimised for both, let alone simultaneous, PET and MRI acquisitions. The development of anthropomorphic phantoms and tissue equivalent materials for PET/MRI has been an active field over the past decade, with an increasing focus toward material characterisation and reproducible manufacture. Further work is required to develop phantoms suitable for holistic performance evaluation of PET/MRI scanners and in establishing robust manufacture techniques accounting for variation in tissue equivalent materials for improved anthropomorphic phantoms.

## Data Availability

Not Applicable
